# Synthesis and Evaluation of Bicyclo[3.1.0]hexane-Based UDP-Gal*f* Analogues as Inhibitors of the Mycobacterial Galactofuranosyltransferase GlfT2

**DOI:** 10.3390/molecules21081053

**Published:** 2016-08-12

**Authors:** Todd L. Lowary, Jing Li

**Affiliations:** 1 Department of Chemistry and Alberta Glycomics Centre, University of Alberta, 11227 Saskatchewan Drive, Edmonton, AB T6G2G2, Canada; tlowary@ualberta.ca; 2 College of Life Science and Technology, Heilongjiang Bayi Agricultural University, Daqing 163319, Heilongjiang Province, China

**Keywords:** mycobacteria, tuberculosis, galactofuranosyltransferases, UDP-Gal*f*, inhibitors, reductive amination

## Abstract

UDP-galactofuranose (UDP-Gal*f*) is the donor substrate for both bifunctional galactofuranosyltransferases, GlfT1 and GlfT2, which are involved in the biosynthesis of mycobacterial galactan. In this paper, a group of UDP-Gal*f* mimics were synthesized via reductive amination of a bicyclo[3.1.0]hexane-based amine by reacting with aromatic, linear, or uridine-containing aldehydes. These compounds were evaluated against GlfT2 using a coupled spectrophotometric assay, and were shown to be weak inhibitors of the enzyme.

## 1. Introduction

*Mycobacterium tuberculosis* and other mycobacterial species have a unique lipidated polysaccharide structure in their cell wall, the mycolyl-arabinogalactan (mAG) complex, which provides the organism with significant protection from the environment [1,2,3,4]. The polysaccharide portion of the mAG complex contains a galactan domain with approximately 30 galactofuranose (Gal*f*) residues attached via alternating β-(1→5) and β-(1→6) linkages. All of the galactose residues in mycobacterial galactan are in the furanose form, an isomer of this monosaccharide that is absent in humans [5]. Thus, the glycosyltransferases that are involved in the biosynthesis of mycobacterial galactan are viewed as potential targets for development of new antibacterial agents [6,7,8].

**Scheme 1:**
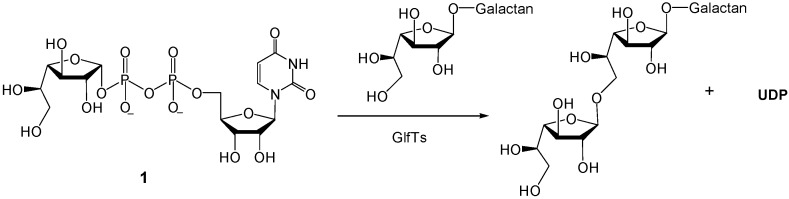
The β-Gal*f*-(1→6)-β-Gal*f* transferase activity of mycobacterial galactofuranosyltransferase GlfT2. Gal*f*: galactofuranose.

Mycobacterial galactan is assembled by the combined action of two bifunctional galactofuranosyltransferases, GlfT1 and GlfT2 [7,9,10]. Both transfer galactofuranose from UDP-Gal*f* (1, Scheme 1), a sugar nucleotide that is produced from UDP-galactopyranose (UDP-Gal*p*) by the action of UDP-Gal*p* mutase [8] to an acceptor oligosaccharide. GlfT1 possesses β-Gal*f*-(1→4)-α-Rha*p* and β-Gal*f*-(1→5)-β-Gal*f* transferase activity, whereas GlfT2 harbors β-Gal*f*-(1→5)-β-Gal*f* and β-Gal*f*-(1→6)-β-Gal*f* transferase activity. The latter of these activities for GlfT2 is shown in Scheme 1.

**Figure 1:**
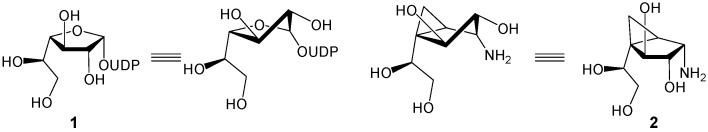
Comparison of the anticipated conformation of **1** with bicyclo[3.1.0]hexane derivative **2**.

In the search for inhibitors of mycobacterial galactosyltransferases and other galactofuranose-recognizing proteins, UDP-Gal*f* analogues have drawn significant attention. Amongst those synthesized, a common structural modification is the decoration of hydroxyl groups on the galactose [11,12] and the replacement of the ring oxygen by other atoms [13,14,15]. In a previous paper, we reported the synthesis of a potential mimetic of **1**, the bicyclo[3.1.0]hexane-based derivative **2** (Figure 1) [16]. Based on previous computational investigations, we anticipated that the five-membered ring in **1** would adopt an envelope conformation in which C-2 was above the plane formed by C-1, O-4, C-4, and C-3 [17,18]. In **2**, the five-membered ring is locked into an envelope in which the cyclopropane methylene group is on the same side of the ring as the flap formed by the cyclopentane carbon [19]. Thus, we hypothesized that compound **2** functionalized on the nitrogen with different groups could mimic **1** and serve as GlfT2 inhibitors. In this paper, we describe an exploration of this hypothesis.

## 2. Results and Discussion

### 2.1. Design Considerations

**Figure 2:**
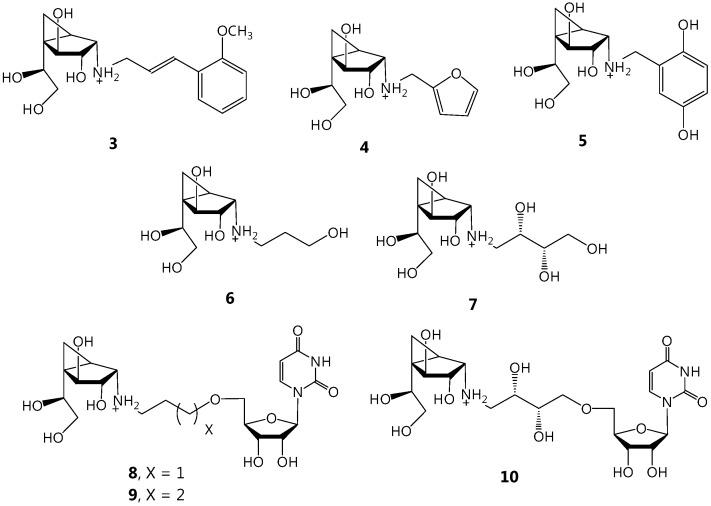
Target mimics of UDP-Gal*f* (**1**), based on the bicyclo[3.1.0]hexane derivative **2**.

As targets, we chose molecules containing different groups that could fill the binding pocket of GlfT2 that would normally be occupied by the uridine diphosphate moiety of **1**. In total, eight compounds (**3**–**10**, Figure 2) were targeted for synthesis. The key step was to use the amino group of **2** in a reductive amination strategy to form the corresponding *N*-alkylated derivatives. The amino group in the compounds was expected to play an important role in the inhibition of the enzyme. Under physiological conditions, this group would be protonated and would thus provide a positive charge close to the sugar ring, which could mimic the anticipated electrophilic transition state of the GlfT2-catalyzed glycosylation.

### 2.2. Synthesis of Target Molecules

**Scheme 2:**
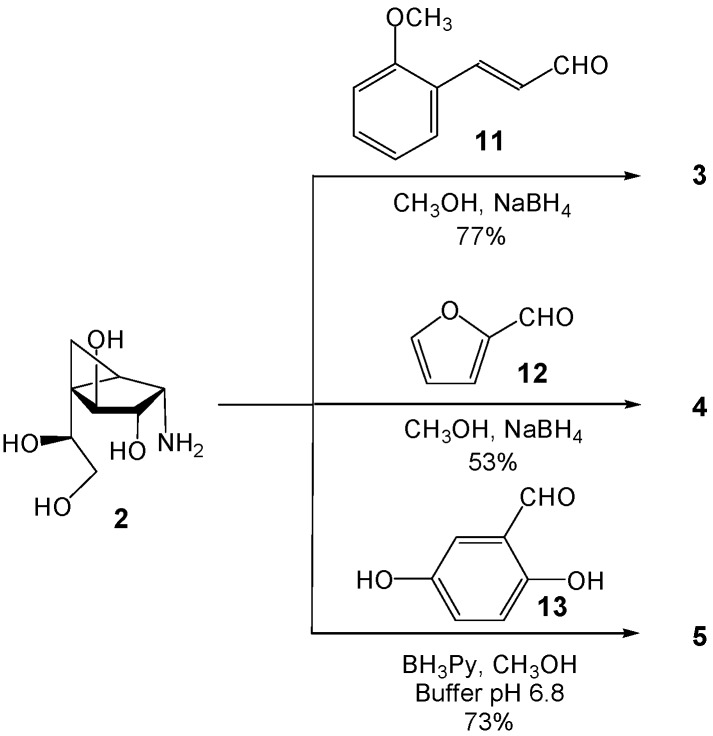
Synthesis of **3**–**5**.

Three analogues (**3**–**5**), containing an aromatic domain, could interact with amino acids in the active site either through cation–π or π–π stacking interactions [20]. To access these molecules (Scheme 2) commercially-available aldehydes **11**, **12**, or **13** were treated with **2** in freshly distilled methanol to form the imines, which were then reduced with either NaBH_4_ or borane–pyridine (BH_3_·Py) complex leading to **3**, **4**, and **5**, respectively. The yields of these reactions were moderate, ranging from 53% to 77%. Normally, NaCNBH_3_ is used in reductive amination reactions [21]; however, NaBH_4_ was used here given its more potent reducing ability of both the imine and the unreacted aldehyde, which minimized the formation of dialkylated compounds. Reductive amination of **13** using BH_3_·Py, gave a better yield than when NaBH_4_ was used as the reducing agent. However, a similar effect was not seen for **11** or **12**; indeed, in the case of **11**, partial reduction of the double bond was observed, as was an increase in the amount of dialkylated byproducts.

In previous molecular modeling studies by van Boom and coworkers [22], a five-atom linker between the uridine and the sugar moiety was shown to provide the required distance to span a pyrophosphate moiety. Hence a group of analogues containing five- or six-member chains attached to the nitrogen were selected for synthesis (**6**–**10**). We chose as targets compound **6**, which has five atoms between the nitrogen and oxygen, and **7**, which has a six-atom linker, but with more hydroxyl groups that might act as the chelating sites to metal ions involved in the transferase reaction [23]. Compounds **8**–**10** contain the uridine moiety, and have five or six atoms between the bicyclohexane moiety and the uridine.

**Scheme 3:**
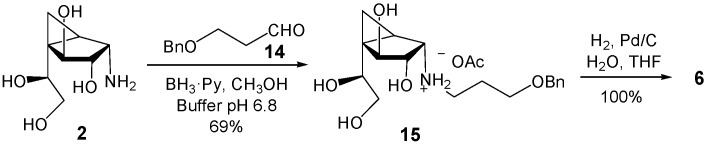
Synthesis of **6**.

The synthesis of **6** is shown in Scheme 3. Aldehyde **14** [24] and **2** were mixed in freshly distilled methanol and deoxygenated phosphate buffer (pH 6.8) and then reacted with BH_3_·Py to afford **15** in 69% yield. The phosphate buffer was added to increase the rate of imine reduction [25]. Solvent deoxygenation was important to prevent N-methylation through aerobic oxidation of methanol to formaldehyde, imine formation, and reduction. Hydrogenolysis of **15** in H_2_O and THF afforded the target **6** in quantitative yield.

**Scheme 4:**
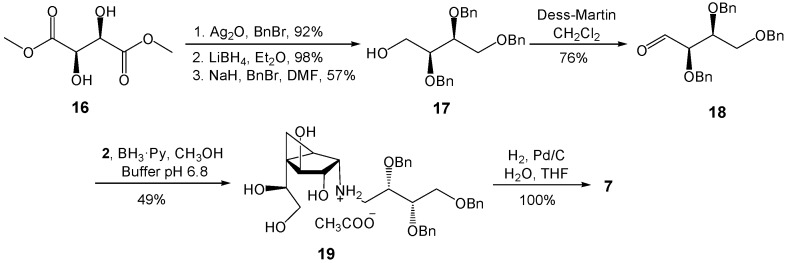
Synthesis of **7**.

To access **7** (Scheme 4) commercially-available 1,4-dimethyl-l-tartrate (**16**) was treated with benzyl bromide and freshly prepared silver oxide to give the expected dibenzyl ether, which was reduced to the corresponding diol with LiBH_4_ in ether; subsequent monobenzylation of the product with sodium hydride and benzyl bromide gave **17** in 51% over the three step sequence [26]. The primary alcohol was oxidized by Dess–Martin periodinane reagent to afford, in 76% yield, aldehyde **18**. The compound was subsequently treated with **2** and BH_3_·Py in methanol and phosphate buffer (pH 6.8) to give a 49% yield of **19**. Finally, target **7** was obtained in quantitative yield by hydrogenolysis over Pd–C in H_2_O and THF.

**Scheme 5:**
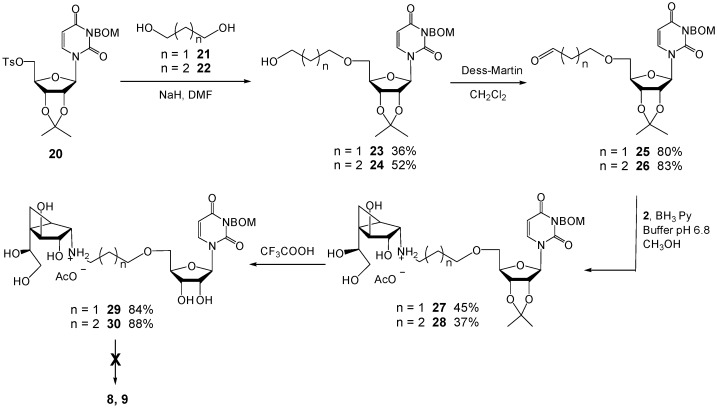
Attempted synthesis of **8** and **9**.

The first step required for the preparation of compounds **8**–**10** was to generate an activated uridine derivative **20** (Scheme 5) [27], which could then be attached to a linker and finally coupled with the bicyclo[3.1.0]hexane amine **2**. Reaction of **20** with 10 equivalents of diol **21** or **22** and 1.2 equivalents of NaH in dimethylformamide (DMF) afforded compounds **23** and **24** in moderate yield (36% and 52%, respectively). Attempts to improve the yield of this transformation by changing the ratio of the starting materials and the sequence of adding the reagents were unsuccessful. These primary alcohols were then oxidized, in ~80% yield, into aldehydes **25** and **26**, which were treated with **2** and BH_3_·Py in methanol and phosphate buffer (pH 6.8) to give the ammonium salts **27** and **28**, respectively. The acetate anion was introduced during work up, which involves acidification with acetic acid. The yield of the reductive amination reaction was low and both the amine and the aldehyde were found unconsumed at the end of the reaction. However, elongation of the reaction time led to the formation of an *N*-methylated byproduct, which was inseparable from the target compound.

With **27** and **28** in hand, the remaining step was to remove both the isopropylidene ketal and the benzyloxymethyl (BOM) aminal. We initially explored hydrogenation to remove the BOM aminal, but treatment of H_2_ in the presence of Pd/C or Pd(OH)_2_ only resulted in starting material being returned. We then explored the use of Lewis acids to cleave both protecting groups. However, when treated with boron trichloride or boron tribromide in a number of solvent systems, none of the desired product was formed. The starting compound was decomposed in the reaction and thin-layer chromatography (TLC) revealed a number of products. However, when **27** and **28** were treated with trifluoroacetic acid (TFA), only the isopropylidene acetal was cleaved and compounds **29** and **30** were obtained in good yield. Given the problems we had in removing the BOM acetal, we chose instead to test compounds **29** and **30** against GlfT2.

**Scheme 6:**
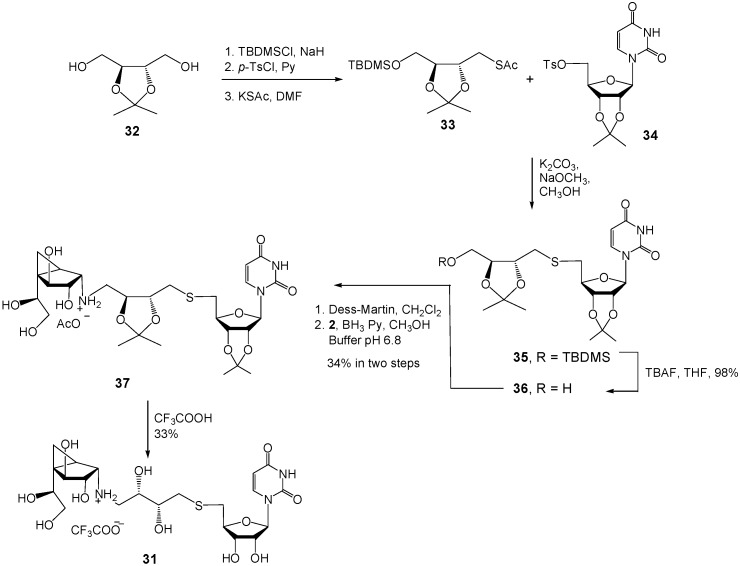
Synthesis of **31**.

We then turned our attention to the synthesis of the final target, **10**. Mindful of the low yields obtained in the alkylation of **20** with diols (synthesis of **23** and **24**), and our inability to remove the BOM aminal from compounds **27** and **28**, we chose to modify the target, by replacing O-5 in the nucleoside domain with a sulfur (derivative **31**, Scheme 6). This approach necessitated the preparation of thioacetate **33** from **32** (Scheme 6). Upon treatment with a weak base, **33** would generate a thiolate that could react with an electrophile. We anticipated that the enhanced nucleophilicity of this thiolate compared to an alkoxide would facilitate the alkylation reaction. Moreover, this modification would require less basic conditions, which would obviate the need for the use of an N-protecting group (i.e., BOM).

In executing this approach, compound **32** underwent monosilylation, tosylation, and displacement with potassium thioacetate to form **33**. This compound was then deacylated and then treated with **34** to produce **35** in 48% over five steps. After desilylation of **35** with tetra-n-butylammonium fluoride (TBAF), alcohol **36** was obtained in 98% yield [28]. Compound **36** was then oxidized with Dess–Martin periodinane in CH_2_Cl_2_ to give the aldehyde, which was coupled, without purification, with **2** and BH_3_·Py to afford a 34% yield **38**. The target **31** was obtained successfully after the cleavage of isopropylidene acetal upon treatment with trifluoroacetic acid in 33% yield. TLC showed complete conversion of the substrates. The poor yield is because of losses during chromatography.

### 2.3. Evaluation of Analogues as Inhibitors of GlfT2

**Figure 3:**
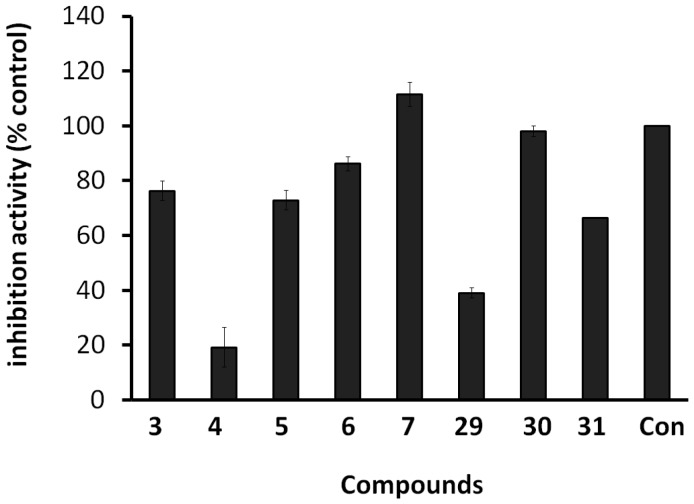
Inhibition activity of the bicyclo[3.1.0]hexane-based derivatives against GlfT2.

These UDP-Galf analogues were investigated as potential inhibitors of mycobacterial GlfT2 using a reported coupled spectrophotometric assay [29]. In these assays, a potential inhibitor is added to the incubation mixture together with a known trisaccharide substrate (β-d-Gal*f*-(1→5)-β-d-Gal*f*-(1→6)-β-d-Gal*f*-Octyl) [30] and UDP-Gal*f* (**1**). All of the UDP-Gal*f* analogues were screened at a concentration of 4 mM against GlfT2 at 37 °C, using 750 µM UDP-Gal*f*. The percentage activities compared to the no inhibitor control are shown in Figure 3. Of all of the compounds, the one incorporating the furan moiety (compound **4**) was the most potent, showing only 18% activity (82% inhibition). The next most potent compound was the BOM-protected derivative **29** which inhibited the enzyme by approximately 50%. All of the other compounds showed less than 40% inhibition; given the concentration at which they were tested compared to the *K_M_* of the **1** (~250 µM) [31], this is reflective of these compounds being poor inhibitors. Compounds **4** and **29** do not share obvious structural similarities, except that both possess a heterocyclic ring. It is therefore difficult to draw conclusions about the enhanced potency of these compounds compared to the others.

## 3. Experimental Section

### 3.1. Synthesis of Target Molecules

**Figure 4:**
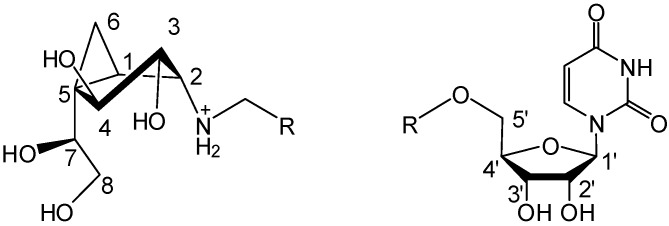
Numbering system used for NMR assignments.

All reagents were purchased from commercial sources and were used without further purification. Solvents used in reactions were pre-dried by PURESOLV-400 System from Innovative Technology Inc. (Amesbury, MA, USA). All reactions were monitored by TLC on silica gel G-25 UV254 (0.25 mm, Macherey–Nagel). Spots were detected under UV light and/or by charring with acidified ethanolic anisaldehyde. Solvents were evaporated under reduced pressure and below 50 °C (water bath). Column chromatography was performed on silica gel 60 (40–60 μm). The ratio between silica gel and crude product ranged from 100:1 to 20:1 (*w*/*w*). Iatrobeads refers to a beaded silica gel 6RS-8060, which was manufactured by Iatron laboratories (Tokyo, Japan). ^1^H-NMR spectra were recorded on VARIAN INOVA-NMR spectrometers (Varian, Inc., Salt Lake City, UT, USA) at 400, 500, or 600 MHz and chemical shifts are referenced to CDCl_3_ (7.26, CDCl_3_) or CD_3_OD (4.78, CD_3_OD). ^13^C-NMR APT spectra were recorded at 100 or 125 MHz, and chemical shifts are referenced to CDCl_3_ (77.23, CDCl_3_) or CD_3_OD (48.9, CD_3_OD). ^1^H-NMR data are reported as though they are first order, and the peak assignments are made by 2D-NMR spectroscopy (^1^H–^1^H COSY and HMQC). The numbering system used for NMR assignment is shown in Figure 4. HRMS-ESI spectra were recorded on samples suspended in THF or CH_3_OH and added NaCl. Optical rotations were measured on Perkin-Elmer 241 Polarimeter (Perkin-Elmer, Waltham, MA, USA) with sodium D line (589 nm) and are in units of deg·mL (dm·g)^−1^.

*(1R,2S,3S,4S,5S)-5-(1,2-Dihydroxyethyl)-3,4-dihydroxy-N-((E)-3-(2-methoxyphenyl)allyl)bicyclo[3.1.0]hexan-2-ammonium acetate* (**3**). A solution of **2** (8.8 mg, 0.046 mmol) and (*E*)-3-(2-methoxyphenyl) acrylaldehyde (**11**, 7.5 mg, 0.046 mmol) in freshly distilled CH_3_OH (2 mL) was stirred at rt for 1 h, before being cooled to −30 °C. NaBH_4_ (5.3 mg, 0.14 mmol) was added, and the solution was stirred for 5 min before being warmed to rt followed by stirring for an additional 10 min. The solution was then acidified with HOAc to pH 5 and concentrated. The resulting residue was purified by chromatography on Iatrobeads (CH_2_Cl_2_–CH_3_OH 10:1→1:10) to give **3** (14 mg, 77%) as a white foam. *R_f_* 0.36 (CH_3_OH–NH_4_OH 40:1); $[\alpha {]}_{D}^{25}$ +31.8 (*c* 1.41, CH_3_OH); ^1^H-NMR (500 MHz, CD_3_OD, δ_H_) 7.47 (dd, 1H, *J* = 1.3, 7.6 Hz, Ar), 7.27 (ddd, 1H, *J* = 1.3, 7.4, 8.6 Hz, Ar), 7.09 (d, 1H, *J* = 16.0 Hz, =CHPh), 6.98 (d, 1H, *J* = 8.4 Hz, Ar), 6.92 (dd, 1H, *J* = 7.4, 7.6 Hz, Ar), 6.33 (ddd, 1H, *J* = 7.0, 7.2, 16.0 Hz, CH_2_CH=), 4.29 (d, 1H, *J* = 6.8 Hz, H-4),3.92 (dd, 1H, *J* = 5.9, 6.3 Hz, H-7), 3.88–3.77 (m, 6H, NHCH_2_, OCH_3_, H-3), 3.67 (dd, 1H, *J* = 5.9, 11.1 Hz, H-8), 3.51 (dd, 1H, *J* = 6.3, 11.2 Hz, H-8), 3.49 (d, 1H, *J* = 6.3 Hz, H-2), 1.76 (dd, 1H, *J* = 3.8, 8.8 Hz, H-1), 0.91 (dd, 1H, *J* = 6.1, 8.8 Hz, H-6), 0.78 (dd, 1H, *J* = 3.8, 6.1 Hz, H-6); ^13^C-NMR (125 MHz, CD_3_OD, δ_C_) 158.5 (Ar), 133.7 (CH=), 130.8 (Ar), 128.4 (Ar), 125.9 (Ar), 121.7 (Ar), 121.5 (CH=), 112.2 (Ar), 79.7 (C-4), 76.7 (C-3), 72.2 (C-7), 66.0 (C-8), 59.9 (C-2), 56.0 (CH_3_), 49.1 (NHCH_2_), 35.7 (C-5), 20.5 (C-1), 8.9 (C-6);HRMS (ESI) *m/z* Calcd for (M − CH_3_COO^−^) C_18_H_26_NO_5_: 336.1805. Found: 336.1806.

*(1R,2S,3S,4S,5S)-5-(1,2-Dihydroxyethyl)-N-(furan-2-ylmethyl)-3,4-dihydroxy-bicyclo[3.1.0]hexan-2-ammonium acetate* **(4)**. A solution of **2** (6.7 mg, 0.035 mmol) and furan-2-carbaldehyde (**12**, 3.4 mg, 0.035 mmol) in freshly distilled CH_3_OH (2 mL) was stirred at rt for 1 h, before being cooled to −30 °C. NaBH_4_ (5.0 mg, 0.13 mmol) was added, and the solution was stirred for 5 min before being warmed to rt followed by stirring for an additional 10 min. The solution was then acidified with HOAc to pH 5 and concentrated. The resulting residue was purified by chromatography on Iatrobeads (CH_2_Cl_2_–CH_3_OH 10:1→1:10) to give **4** (6.2 mg, 53%) as a white foam. *R_f_* 0.27 (pure CH_3_OH); $[\alpha {]}_{D}^{25}$ +55.0 (*c* 0.35, CH_3_OH); ^1^H-NMR (400 MHz, CD_3_OD, δ_H_) 7.43 (d, 1H, *J* = 1.9 Hz, furan CH), 6.34 (dd, 1H, *J* = 1.9, 3.2 Hz, furan CH), 6.26 (d, 1H, *J* = 3.2 Hz, furan CH), 4.19 (d, 1H, *J* = 6.9 Hz, H-4), 3.87 (d, 1H, *J* = 14.4 Hz, NCH_2_), 3.79 (dd, 1H, *J* = 6.1, 6.3 Hz, H-7), 3.76 (d, 1H, *J* = 11.4 Hz, NCH_2_), 3.65 (dd, 1H, *J* = 6.1, 11.1 Hz, H-8), 3.59 (dd, 1H, *J* = 6.2, 6.9 Hz, H-3), 3.49 (dd, 1H, *J* = 6.3, 11.1 Hz, H-8), 2.99 (d, 1H, *J* = 6.2 Hz, H-2), 1.50 (dd, 1H, *J* = 5.1, 7.8 Hz, H-1), 0.68–0.66 (m, 2H, 2 × H-6); ^13^C-NMR (100 MHz, CD_3_OD, δ_C_) 154.5 (C=CH), 143.3 (furan CH), 111.2 (furan CH), 108.4 (furan CH), 80.1 (C-4), 77.7 (C-3), 73.3 (C-7), 66.1 (C-8), 59.2 (C-2), 45.1 (NCH_2_), 34.4 (C-5), 23.9 (C-1), 8.6 (C-6); HRMS (ESI) *m/z* Calcd for (M − CH_3_COO^−^) C_13_H_20_NO_5_: 270.1336. Found: 270.1337.

*(1R,2S,3S,4S,5S)-N-(2,5-Dihydroxybenzyl)-5-(1,2-dihydroxyethyl)-3,4-dihy-droxybicyclo[3.1.0]hexan-2-ammonium acetate* (**5**). A solution of **2** (11 mg, 0.053 mmol) and 2,5-dihydroxybenzaldehyde (**13**, 7.3 mg, 0.053 mmol) in freshly distilled CH_3_OH (2 mL) was stirred at rt for 1 h. To this mixture was added phosphate buffer (0.1 M, pH 6.8, 0.2 mL) and the solution was cooled to 0 °C before BH_3_·pyridine (20 mL, 0.16 mmol) was added. The reaction mixture was then warmed to rt and stirred overnight before being acidified with HOAc to pH 5 and concentrated. The resulting residue was purified by chromatography on Iatrobeads (CH_2_Cl_2_–CH_3_OH 10:1→1:10) to give compound 5 (14.4 mg, 73%) as a white foam. *R_f_* 0.46 (CH_3_OH–NH_4_OH 20:1); $[\alpha {]}_{D}^{25}$ +31.5 (*c* 0.32, CH_3_OH); ^1^H-NMR (500 MHz, CD_3_OD, δ_H_) 6.77 (d, 1H, *J* = 2.9 Hz, Ar), 6.71 (d, 1H, *J* = 8.6 Hz, Ar), 6.66 (dd, 1H, *J* = 2.9, 8.6 Hz, Ar), 4.29 (d, 1H, *J* = 7.0 Hz, H-4),4.22 (d, 1H, *J* = 13.2 Hz, CH_2_N), 4.09 (d, 1H, *J* = 13.2 Hz, CH_2_N), 3.97 (dd, 1H, *J* = 5.7, 6.5 Hz, H-7), 3.76 (dd, 1H, *J* = 6.6, 7.0 Hz, H-3), 3.66 (dd, 1H, *J* = 5.7, 11.1 Hz, H-8), 3.66 (dd, 1H, *J* = 6.5, 11.1 Hz, H-8), 3.29 (d, 1H, *J* = 6.6 Hz, H-2),1.79 (dd, 1H, *J* = 4.0, 8.6 Hz, H-1), 0.84 (dd, 1H, *J* = 5.9, 8.6 Hz, H-6), 0.73 (dd, 1H, *J* = 4.0, 5.9 Hz, H-6); ^13^C-NMR (125 MHz, CD_3_OD, δ_C_) 151.5 (Ar), 150.4 (Ar), 121.3 (Ar), 118.4 (Ar), 117.8 (Ar), 117.3 (Ar), 79.6 (C-4), 76.5 (C-3), 72.1 (C-7), 66.0 (C-8), 59.5 (C-2), 48.1 (CH_2_N), 35.5 (C-5), 20.8 (C-1), 8.6 (C-6). HRMS (ESI) *m/z* Calcd for (M − CH_3_COO^−^) C_15_H_22_NO_6_: 312.1442. Found: 312.1442.

*(1R,2S,3S,4S,5S)-N-(3-(Benzyloxy)propyl)-5-(1,2-dihydroxyethyl)-3,4-dihy-droxybicyclo[3.1.0]hexan-2-ammonium acetate* (**15**). A solution of **2** (9.0 mg, 0.047 mmol) and **14** (7.8 mg, 0.047 mmol) in fresh CH_3_OH (2 mL) was stirred at rt for 1 h. To this mixture was added phosphate buffer (0.1 M, pH 6.8, 0.2 mL) and the solution was cooled to 0 °C before BH_3_·pyridine (20 mL, 0.16 mmol) was added. The reaction mixture was then warmed to rt and stirred overnight before being acidified with HOAc to pH 5 and concentrated. The resulting residue was purified by chromatography on Iatrobeads (CH_2_Cl_2_–CH_3_OH from 10:1 to 1:10) to give **15** (13.4 mg, 69%) as a white foam. *R_f_* 0.48 (CH_3_OH–NH_4_OH 20:1); $[\alpha {]}_{D}^{25}$ +15.4 (*c* 0.47, CH_3_OH); ^1^H-NMR (500 MHz, CD_3_OD, δ_H_) 7.35–7.24 (m, 5H, Ar), 4.54 (d, 1H, *J* = 11.9 Hz, CH_2_Ph), 4.53 (d, 1H, *J* = 11.9 Hz, CH_2_Ph), 4.26 (d, 1H, *J* = 6.8 Hz, H-4), 3.94 (dd, 1H, *J* = 6.4, 6.2 Hz, H-7), 3.83 (dd, 1H, *J* = 6.4, 6.8 Hz, H-3), 3.67–3.60 (m, 3H, H-8, CH_2_OBn), 3.50 (dd, 1H, *J* = 6.4, 11.1 Hz, H-8), 3.46 (d, 1H, *J* = 6.4 Hz, H-2), 3.33–3.29 (m, 1H, 1 × NHCH_2_), 3.10 (ddd, 1H, *J* = 7.2, 7.2, 12.3 Hz, NHCH_2_), 2.11–1.97 (m, 4H, CH_2_CH_2_), 1.73 (dd, 1H, *J* = 4.0, 8.8 Hz, H-1), 0.88 (dd, 1H, *J* = 5.7, 8.8 Hz, H-6), 0.75 (dd, 1H, *J* = 4.0, 5.7 Hz, H-6); ^13^C-NMR (125 MHz, CD_3_OD, δ_C_) 139.3 (Ar), 129.5 (2 C, Ar × 2), 129.1 (2 C, Ar × 2), 128.8 (Ar), 79.6 (C-4), 76.5 (C-3), 74.3 (CH_2_Ph), 71.9 (C-7), 69.5 (CH_2_OBn), 66.0 (C-8), 60.9 (C-2), 46.5 (NHCH_2_), 35.9 (C-5), 27.6 (2 C, CH_2_CH_2_), 20.2 (C-1), 8.6 (C-6). HRMS (ESI) *m/z* Calcd for (M − CH_3_COO^−^) C_18_H_28_NO_5_: 338.1962. Found: 338.1961.

*(1R,2S,3S,4S,5S)-5-(1,2-Dihydroxyethyl)-3,4-dihydroxy-N-(3-hydroxypropyl)-bicyclo[3.1.0]hexan-2-ammonium acetate* (**6**). To a solution of compound **15** (13.4 mg, 0.034 mmol) in THF (4 mL) and H_2_O (0.5 mL) was added 10% Pd–C (4 mg), and the mixture was stirred under an H_2_ atmosphere for 12 h. The mixture was then filtered through Celite and concentrated. The resulting crude residue was purified by chromatography on Iatrobeads (CH_2_Cl_2_–CH_3_OH 10:1→100% CH_3_OH) to yield product 6 (10.3 mg, 100%) as a colorless oil. *R_f_* 0.18 (CH_3_OH–NH_4_OH 20:1); $[\alpha {]}_{D}^{25}$ +23.4 (*c* 0.28, CH_3_OH); ^1^H-NMR (400 MHz, CD_3_OD, δ_H_) 4.28 (d, 1 H, *J* = 6.8 Hz, H-4), 3.93 (dd, 1H, *J* = 5.9, 6.3 Hz, H-7), 3.81 (dd, 1H, *J* = 6.3, 6.8 Hz, H-3),3.77–3.70 (m, 2H, CH_2_CH_2_OH), 3.66 (dd, 1H, *J* = 5.9, 11.1 Hz, H-8), 3.50 (dd, 1H, *J* = 6.3, 11.1 Hz, H-8), 3.44 (d, 1H, *J* = 6.3 Hz, H-2), 3.33–3.29 (m, 1H, 1 × NHCH_2_), 3.07 (ddd, 1H, *J* = 7.1, 7.1, 12.3 Hz, NHCH_2_), 1.99–1.86 (m, 2H, CH_2_), 1.73 (dd, 1H, *J* = 4.0, 8.8 Hz, H-1), 0.89 (dd, 1H, *J* = 5.7, 8.8 Hz, H-6), 0.76 (dd, 1H, *J* = 4.0, 5.7 Hz, H-6); ^13^C-NMR (125 MHz, CD_3_OD, δ_C_) 79.6 (C-4), 76.5 (C-3), 72.1 (C-7), 65.9 (C-8), 61.2 (CH_2_OH), 60.8 (C-2), 46.4 (NHCH_2_), 35.7 (C-5), 29.9 (2 C, CH_2_CH_2_), 20.5 (C-1), 8.7 (C-6). HRMS (ESI) *m/z* Calcd for (M − CH_3_COO^−^) C_11_H_22_NO_5_: 248.1492. Found: 248.1493.

*(2S,3S)-2,3,4-Tris(benzyloxy)butan-1-ol* (**17**). To a solution of (2*S*,3*S*)-2,3-bis(benzyloxy)butane-1,4-diol (**16**, 0.23 g, 0.76 mmol) and benzyl bromide (0.13 g, 0.76 mmol) in DMF (4 mL) at 0 °C was added NaH (30 mg, 0.76 mmol, 60% in mineral oil). After stirring for 1 h, the reagents were quenched by the addition of H_2_O. The solution was extracted with Et_2_O twice and the organic layer was washed with brine, dried (MgSO_4_), and concentrated. The residue was purified by chromatography (EtOAc–Hexane 1:4) to give **17** (0.17 g, 57%) as a colorless oil. *R_f_* 0.30 (EtOAc–Hexane 1:2); $[\alpha {]}_{D}^{25}$ +12.3 (*c* 1.31, CH_2_Cl_2_); ^1^H-NMR (500 MHz, CDCl_3_, δ_H_) 7.38–7.28 (m, 15 H, Ar), 4.74 (d, 1H, *J* = 11.8 Hz, CH_2_Ph), 4.70–4.62 (m, 3H, 3 × CH_2_Ph), 4.54 (s, 2H, 2 × CH_2_Ph), 3.84–3.63 (m, 6 H, 2 × CHOBn, 2 × CH_2_OBn, 2 × CH_2_OH), 2.23 (dd, 1H, *J* = 5.4, 7.0 Hz, OH); ^13^C-NMR (125 MHz, CDCl_3_, δ_C_) 138.3 (2 C, Ar × 2), 137.9 (Ar), 128.5 (2 C, Ar × 2), 128.4 (2 C, Ar × 2), 128.3 (2 C, Ar × 2), 128.0 (2 C, Ar × 2), 127.9 (2 C, Ar × 2), 127.8 (2 C, Ar × 2), 127.7 (3 C, Ar × 3), 79.2 (CHOBn), 78.5 (CHOBn), 73.5 (CH_2_Ph), 72.9 (CH_2_Ph), 72.8 (CH_2_Ph), 69.5 (CH_2_OBn), 61.5 (CH_2_OH). HRMS (ESI) *m/z* Calcd for (M + Na^+^) C_25_H_28_O_4_: 415.1880. Found: 415.1876.

*(2R,3S)-2,3,4-Tris(benzyloxy)butanal* (**18**). To a solution of **17** (52 mg, 0.13 mmol) in CH_2_Cl_2_ (2 mL) at 0 °C was added a solution of Dess–Martin periodinane (56 mg, 0.13 mmol) in CH_2_Cl_2_ (2 mL). The mixture was stirred at 0 °C for 2 h and then poured into a cold aqueous saturated NaHCO_3_ solution. The organic layer was washed with brine, dried (MgSO_4_), concentrated, and the residue was purified by chromatography (EtOAc–Hexane 1:6) to give **18** (39 mg, 76%) as a colorless oil. *R_f_* 0.43 (EtOAc–Hexane 1:3); $[\alpha {]}_{D}^{25}$ +3.3 (*c* 0.65, CH_2_Cl_2_); ^1^H-NMR (400 MHz, CDCl_3,_ δ_H_) 9.71 (s, 1H, CHO), 7.37–7.26 (m, 15H, Ar), 4.76 (d, 1H, *J* = 12.0 Hz, CH_2_Ph), 4.64 (d, 1H, *J* = 11.9 Hz, CH_2_Ph), 4.57 (d, 1H, *J* = 12.0 Hz, CH_2_Ph), 4.56 (d, 1H, *J* = 11.9 Hz, CH_2_Ph), 4.48 (d, 1H, *J* = 12.0 Hz, CH_2_Ph), 4.46 (d, 1H, *J* = 12.0 Hz, CH_2_Ph), 4.00–3.96 (m, 2H, 2 × CHOBn), 3.71–3.64 (m, 2H, 2 × CH_2_OBn); ^13^C-NMR (100 MHz, CDCl_3_, δ_C_) 202.38 (CHO), 137.8 (Ar), 137.7 (Ar), 137.2 (Ar), 128.5 (2C, Ar × 2), 128.4 (4C, Ar × 4), 128.2 (2C, Ar × 2), 128.1 (Ar), 128.0 (2C, Ar × 2), 127.8 (Ar), 127.7 (3C, Ar × 3), 82.8 (CHOBn), 77.9 (CHOBn), 73.4 (2C, 2 × CH_2_Ph), 72.9 (CH_2_Ph), 68.1 (CH_2_OBn). HRMS (ESI) *m/z* Calcd for (M + Na^+^) C_25_H_26_O_4_: 413.1679. Found: 413.1674.

*(1R,2S,3S,4S,5S)-5-(1,2-Dihydroxyethyl)-3,4-dihydroxy-N-((2S,3S)-2,3,4-tris-(benzyloxy)butyl)bicycle[3.1.0]hexan-2-ammonium acetate* (**19**). A solution of **2** (11.6 mg, 0.05 mmol) and **18** (21 mg, 0.05 mmol) in freshly distilled CH_3_OH (2 mL) was stirred at rt for 1 h. To this mixture was added phosphate buffer (0.1 M, pH 6.8, 0.2 mL) and the solution was cooled to 0 °C, before and BH_3_·pyridine (20 mL, 0.16 mmol) was added. The reaction mixture was then warmed to rt and stirred overnight before being acidified with HOAc to pH 5 and then concentrated. The resulting residue was purified by chromatography on Iatrobeads (CH_2_Cl_2_–CH_3_OH 10:1 to 1:1) to give **19** (14.2 mg, 49%) as a white foam. *R_f_* 0.18 (CH_3_OH–CH_2_Cl_2_ 1:1); $[\alpha {]}_{D}^{25}$ +16.5 (*c* 0.39, CH_3_OH); ^1^H-NMR (500 MHz, CD_3_OD, δ_H_) 7.35–7.24 (m, 15H, Ar), 4.68 (d, 1H, *J* = 11.7 Hz, CH_2_Ph), 4.64 (d, 1H, *J* = 11.3 Hz, CH_2_Ph),4.62 (d, 1H, *J* = 11.7 Hz, CH_2_Ph),4.57 (d, 1H, *J* = 11.7 Hz, CH_2_Ph), 4.51–4.50 (m, 2H, 2 × CH_2_Ph), 4.19 (d, 1H, *J* = 7.0 Hz, H-4), 3.86–3.81 (m, 2H, 2 × CHOBn), 3.78 (dd, 1H, *J* = 6.2, 6.2 Hz, H-7), 3.71 (dd, 1H, *J* = 3.1, 10.5 Hz, CH_2_OBn), 3.66–3.59 (m, 3H, H-3, 1 × CH_2_OBn, 1 × H-8), 3.49 (dd, 1H, *J* = 6.2, 11.1 Hz, H-8), 3.07 (dd, 1H, *J* = 2.6, 12.1 Hz, NHCH_2_), 2.99 (d, 1H, *J* = 6.3 Hz, H-2), 2.64 (dd, 1H, *J* = 8.3, 12.1 Hz, NHCH_2_), 1.50 (dd, 1H, *J* = 4.4, 8.6 Hz, H-1), 0.72 (dd, 1H, *J* = 5.8, 8.6 Hz, H-6), 0.68 (dd, 1H, *J* = 4.4, 5.8 Hz, H-6); ^13^C-NMR (125 MHz, CD_3_OD, δ_C_) 139.7 (Ar), 139.5 (2C, Ar × 2), 129.5 (2C, Ar × 2), 129.4 (4C, Ar × 4), 129.3 (2C, Ar × 2), 129.2 (2C, Ar × 2), 129.0 (2C, Ar × 2), 128.9 (Ar), 128.8 (Ar), 128.7 (Ar), 80.0 (C-4), 79.5 (CHOBn), 79.2 (C-3), 77.5 (CHOBn), 74.4 (CH_2_Ph), 74.3 (CH_2_Ph), 73.8 (CH_2_Ph), 73.1 (C-7), 70.2 (CH_2_OBn), 66.1 (C-8), 61.2 (C-2), 49.3 (CH_2_NH), 34.8 (C-5), 23.4 (C-1), 8.8 (C-6). HRMS (ESI) *m/z* Calcd for (M − CH_3_COO^−^) C_33_H_42_NO_7_: 564.2956. Found: 564.2951.

*(1R,2S,3S,4S,5S)-5-(1,2-Dihydroxyethyl)-3,4-dihydroxy-N-((2S,3S)-2,3,4-tri-hydroxybutyl)bicyclo[3.1.0]hexan-2-ammonium acetate* (**7**). To a solution of **19** (20 mg, 0.032 mmol) in THF (4 mL) and H_2_O (0.5 mL) was added 10% Pd–C (4 mg) and the reaction mixture was stirred under a H_2_ atmosphere for 12 h. The mixture was then filtered through Celite and concentrated to give pure **7** (11 mg, 100%) as a colorless oil. *R_f_* 0.28 (CH_3_OH–NH_4_OH 20:1); $[\alpha {]}_{D}^{25}$ +18.9 (*c* 0.24, CH_3_OH); ^1^H-NMR (500 MHz, CD_3_OD, δ_H_) 4.26 (d, 1H, *J* = 6.8 Hz, H-4), 3.97 (ddd, 1H, *J* = 3.3, 3.3, 8.2 Hz, NHCH_2_CHOH), 3.89 (dd, 1H, *J* = 6.2, 6.2 Hz, H-7), 3.83 (dd, 1H, *J* = 6.4, 6.8 Hz, H-3), 3.67–3.58 (m, 4H, CHOHCH_2_OH, 1 × H-8), 3.52 (dd, 1H, *J* = 6.3, 11.1 Hz, H-8), 3.17 (d, 1H, *J* = 6.4 Hz, H-2), 3.32 (dd, 1H, *J* = 3.3, 12.4 Hz, NHCH_2_), 3.10 (dd, 1H, *J* = 8.2, 12.4 Hz, NHCH_2_), 1.71 (dd, 1H, *J* = 4.0, 8.9 Hz, H-1), 0.90 (dd, 1H, *J* = 5.7, 8.9 Hz, H-6), 0.78 (dd, 1H, *J* = 4.0, 5.7 Hz, H-6); ^13^C-NMR (125 MHz, CD_3_OD, δ_C_) 79.7 (C-4), 76.2 (C-3), 74.4 (CHOH), 72.2 (CHOH), 68.8 (CHOH), 65.8 (CH_2_OH), 63.6 (CH_2_OH), 61.4 (C-2), 50.6 (CH_2_NH), 35.7 (C-5), 20.9 (C-1), 8.9 (C-6). HRMS (ESI) *m/z* Calcd for (M − CH_3_COO^−^) C_12_H_24_NO_7_: 294.1547. Found: 294.1543.

*2′,3′-O-Isopropylidene-5′-O-tosyl-3-(benzyloxymethyl)uridine* (**20**). To a solution of 2′,3′-*O*-isopropylidene-3-(benzyloxylmethyl)uridine (4.0 g, 10 mmol) in pyridine (25 mL) was added p-toluenesulfonyl chloride (2.26 g, 12 mmol) at rt. The reaction was stirred overnight and then the excess reagents were quenched by the addition of CH_3_OH (3 mL). The solution was then concentrated and the residue was purified by chromatography (EtOAc–Hexane 1:2) to give **20** (3.94 g, 71%) as a foam. *R_f_* 0.54 (EtOAc–Hexane 1:1); $[\alpha {]}_{D}^{25}$ +23.6 (*c* 1.33, CH_2_Cl_2_); ^1^H-NMR (500 MHz, CDCl_3_, δ_H_) 7.77–7.75 (m, 2H, Ar), 7.37–7.25 (m, 7H, Ar), 7.16 (d, 1H, *J* = 8.1 Hz, CH=), 5.71 (d, 1H, *J* = 8.1 Hz, CH=), 5.60 (d, 1H, *J* = 2.0 Hz, H-1), 5.46 (d, 1H, *J* = 9.8 Hz, NCH_2_OBn), 5.35 (d, 1H, *J* = 9.8 Hz, NCH_2_OBn), 4.88 (dd, 1H, *J* = 2.0, 6.4 Hz, H-2), 4.79 (dd, 1H, *J* = 3.7, 6.4 Hz, H-3), 4.68 (s, 2H, CH_2_Ph), 4.35 (ddd, 1H, *J* = 3.7, 4.6, 4.6 Hz, H-4), 4.27 (d, 2H, *J* = 4.6 Hz, 2 × H-5), 2.42 (s, 3H, CH_3_), 1.55 (s, 3H, CH_3_), 1.34 (s, 3H, CH_3_); ^13^C-NMR (125 MHz, CDCl_3_, δ_C_) 162.3 (C=O), 150.7 (C=O), 145.2 (Ar), 140.6 (CH=), 137.8 (Ar), 132.6 (Ar), 129.9 (2C, Ar × 2), 128.3 (2C, Ar × 2), 127.9 (2C, Ar × 2), 127.7 (Ar), 127.6 (2C, Ar × 2), 114.5 (C(CH_3_)_2_), 102.3 (CH=), 95.6 (C-1), 85.1 (C-4), 84.4 (C-2), 80.8 (C-3), 72.4 (CH_2_Ph), 70.3 (NCH_2_OBn), 69.2 (C-5), 27.0 (CH_3_), 25.2 (CH_3_), 21.6 (CH_3_). HRMS (ESI) *m/z* Calcd for (M + Na^+^) C_27_H_30_N_2_O_9_S: 581.1562. Found: 581.1558.

*2′,3′-O-Isopropylidene-5′-O-(3-hydroxylpropyl)-3-(benzyloxymethyl)uridine* (**23**). To a solution of **20** (0.50 g, 0.9 mmol) and 1,3-propanediol (**21**) (0.69 g, 9.0 mmol) in DMF (4 mL) at 0 °C was added NaH (72 mg, 1.8 mmol, 60% in mineral oil). The solution was stirred at rt for 20 h and then H_2_O (10 mL) was added and the mixture was extracted with Et_2_O. The organic layer was washed with brine, dried (MgSO_4_), and concentrated. The residue was purified by chromatography (EtOAc–Hexane 2:1) to give **23** (0.15 g, 36%) as a colorless oil. *R_f_* 0.14 (EtOAc–Hexane 2.5:1); $[\alpha {]}_{D}^{25}$ −3.3 (*c* 0.96, CH_2_Cl_2_); ^1^H-NMR (400 MHz, CDCl_3_, δ_H_) 7.44 (d, 1H, *J* = 8.2 Hz, CH=), 7.38–7.23 (m, 5H, Ar), 5.77 (d, 1H, *J* = 1.9 Hz, H-1), 5.73 (d, 1H, *J* = 8.2 Hz, CH=), 5.50 (d, 1H, *J* = 9.7 Hz, NCH_2_OBn), 5.47 (d, 1H, *J* = 9.7 Hz, NCH_2_OBn), 4.81–4.77 (m, 2H, H-2, H-3), 4.70 (s, 2H, CH_2_Ph), 4.37 (ddd, 1H, *J* = 3.0, 3.0, 4.5 Hz, H-4),3.73–3.69 (m, 3H, H-5, CH_2_OH), 3.67–3.58 (m, 3H, H-5, CH_2_O), 1.83–1.77 (m, 2H, CH_2_), 1.58 (s, 3H, CH_3_), 1.37 (s, 3H, CH_3_); ^13^C-NMR (125 MHz, CDCl_3_, δ_C_) 162.6 (C=O), 150.8 (C=O), 139.9 (CH=), 137.9 (Ar), 128.3 (2C, Ar × 2), 127.6 (3C, Ar × 3), 114.1 (C(CH_3_)_2_), 101.6 (CH=), 94.4 (C-1), 85.9 (C-2), 85.2 (C-4), 80.9 (C-3), 72.3 (CH_2_Ph), 70.9 (NCH_2_OBn), 70.3 (C-5), 69.6 (CH_2_OCH_2_), 60.6 (CH_2_OH), 32.2 (CH_2_), 27.2 (CH_3_), 25.4 (CH_3_). HRMS (ESI) *m/z* Calcd for (M + Na^+^) C_23_H_30_N_2_O_8_: 485.1894. Found: 485.1894.

*2’,3’-O-Isopropylidene-5’-O-(3-oxopropyl)-3-(benzyloxymethyl)uridine* (**25**). A solution of **23** (32 mg, 0.07 mmol) in CH_2_Cl_2_ (2 mL) was added to a solution of Dess–Martin periodinane (35 mg, 0.08 mmol) in CH_2_Cl_2_ (4 mL) at 0 °C. The reaction was stirred for 3 h at 0 °C and poured into an ice-cold saturated aqueous NaHCO_3_ solution. The organic layer was washed with H_2_O, brine, dried (MgSO_4_), and concentrated. The residue was purified by chromatography (EtOAc–Hexane 1:1) to give **25** (25 mg, 80%) as a colorless oil. *R_f_* 0.26 (EtOAc–Hexane 2:1); $[\alpha {]}_{D}^{25}$ −12.6 (*c* 0.68, CH_2_Cl_2_); ^1^H-NMR (400 MHz, CDCl_3_, δ_H_) 9.75 (dd, 1H, *J* = 1.6, 1.6 Hz, CHO), 7.38 (d, 1H, *J* = 8.2 Hz, CH=), 7.36–7.23 (m, 5H, Ar), 5.81 (d, 1H, *J* = 2.3 Hz, H-1), 5.75 (d, 1H, *J* = 8.2 Hz, CH=), 5.50 (d, 1H, *J* = 9.7 Hz, NCH_2_OBn), 5.46 (d, 1H, *J* = 9.7 Hz, NCH_2_OBn), 4.75 (dd, 2H, *J* = 3.2, 6.2 Hz, H-3), 4.74 (dd, 2H, *J* = 2.3, 6.2 Hz, H-2), 4.70 (s, 2H, CH_2_Ph), 4.35 (ddd, 1H, *J* = 2.8, 3.2, 4.2 Hz, H-4), 3.82–3.79 (m, 2H, CH_2_OCH_2_), 3.74 (dd, 1H, *J* = 2.8, 10.6 Hz, H-5), 3.62 (dd, 1H, *J* = 4.2, 10.6 Hz, H-5), 2.69–2.65 (m, 2H, CH_2_), 1.58 (s, 3H, CH_3_), 1.36 (s, 3H, CH_3_); ^13^C-NMR (100 MHz, CDCl_3_, δ_C_) 199.9 (CHO), 162.6 (C=O), 150.9 (C=O), 139.6 (CH=), 137.9 (Ar), 128.3 (2C, Ar × 2), 127.6 (3C, Ar × 3), 114.1 (C(CH_3_)_2_), 101.7 (CH=), 93.9 (C-1), 85.7 (C-4), 85.2 (C-2), 80.7 (C-3), 72.3 (CH_2_Ph), 71.0 (CH_2_OBn), 70.3 (C-5), 64.8 (CH_2_OCH_2_), 43.7 (CH_2_), 27.2 (CH_3_), 25.3 (CH_3_). HRMS (ESI) *m/z* Calcd for (M + Na^+^) C_23_H_28_N_2_O_8_: 483.1738. Found: 483.1838.

*(1R,2S,3S,4S,5S)-N-(3-(2′,3′-O-Isopropylidene-3-(benzyloxymethyl)uridin)-propyl)-5-(1,2-dihydroxyethyl)-3,4-dihydroxybicyclo[3.1.0]hexan-2-ammonium acetate* (**27**). To a mixture of **2** (8 mg, 0.043 mmol) and **25** (20 mg, 0.043 mmol) in freshly distilled CH_3_OH (2 mL) at 0 °C was added BH_3_·pyridine (20 mL, 0.16 mmol) and phosphate buffer (0.1 M, pH 6.8, 0.4 mL). The mixture was stirred overnight at rt and then acidified with HOAc to pH 5 before being concentrated. The resulting residue was purified by chromatography on C_18_ silica gel (H_2_O–CH_3_OH 10:1→1:10) to give **27** (13.3 mg, 45%) as a white foam. *R_f_* 0.11 (pure CH_3_OH; C_18_ silica gel TLC); $[\alpha {]}_{D}^{25}$ +15.4 (*c* 1.32, CH_3_OH); ^1^H-NMR (300 MHz, CD_3_OD, δ_H_) 7.71 (d, 1H, *J* = 8.1 Hz, CH=), 7.37–7.21 (m, 5H, Ar), 5.80 (d, 1H, *J* = 1.4 Hz, H-1′), 5.73 (d, 1H, *J* = 8.1 Hz, CH=),5.45 (s, 2H, NCH_2_OBn), 4.81 (m, 2H, H-2′, H-3′), 4.66 (s, 2H, CH_2_Ph), 4.38–4.37 (m, 1H, H-4′), 4.17 (d, 1H, *J* = 6.9 Hz, H-4), 3.75–3.47 (m, 8H, H-3, H-7, H-8, H-8, H-5’, H-5’, CH_2_OC-5′), 2.91 (d, 1H, *J* = 6.1 Hz, H-2), 2.80 (ddd, 1H, *J* = 6.8, 7.0, 11.6 Hz, NHCH_2_), 2.56 (ddd, 1H, *J* = 7.0, 7.1, 11.6 Hz, NHCH_2_), 1.79–1.71 (m, 2H, CH_2_), 1.54 (s, 3H, CH_3_), 1.49 (dd, 1H, *J* = 6.0, 6.0 Hz, H-1), 1.36 (s, 3H, CH_3_), 0.68–0.67 (m, 2H, 2 × H-6); ^13^C-NMR (125 MHz, CD_3_OD, δ_C_) 164.9 (C=O), 152.3 (C=O), 142.3 (CH=), 139.5 (Ar), 129.3 (2C, Ar × 2), 128.7 (Ar), 128.6 (2C, Ar × 2), 114.8 (C(CH_3_)_2_), 101.7 (CH=), 95.6 (C-1′), 87.6 (C-4′), 86.6 (C-2′), 82.6 (C-3′), 80.0 (C-4), 77.7 (C-3), 73.3 (C-7), 73.2 (CH_2_Ph), 72.0 (C-5′ or CH_2_OCH_2_), 71.6 (NCH_2_OBn), 70.8 (C-5’ or CH_2_OCH_2_), 66.1 (C-8), 60.7 (C-2), 46.6 (NHCH_2_), 34.5 (C-5), 30.6 (CH_2_), 27.5 (CH_3_), 25.5 (CH_3_), 24.3 (C-1), 8.9 (C-6). HRMS (ESI) *m/z* Calcd for (M − CH_3_COO^−^) C_31_H_44_N_3_O_11_: 634.2970. Found: 634.2969.

*(1R,2S,3S,4S,5S)-N-(3-(3-(Benzyloxymethyl)uridin)propyl)-5-(1,2-dihydroxy-ethyl)-3,4-dihydroxybicyclo[3.1.0]hexan-2-ammonium 2,2,2-trifluoroacetate* (**29**). Compound **27** (14 mg, 0.02 mmol) was dissolved in TFA (2 mL) at 0 °C. The solution was then stirred at rt for 12 h and concentrated. The resulting residue was purified by chromatography on Iatrobeads (CH_2_Cl_2_–CH_3_OH 10:1→1:3) to give the **29** (12 mg, 84%) as a white foam. *R_f_* 0.24 (CH_3_OH–NH_4_OH 40:1); $[\alpha {]}_{D}^{25}$ +14.9 (*c* 0.15, CH_3_OH); ^1^H-NMR (400 MHz, CD_3_OD, δ_H_) 7.76 (d, 1H, *J* = 8.2 Hz, CH=), 7.33–7.24 (m, 5H, Ar), 5.78 (d, 1H, *J* = 2.8 Hz, H-1′), 5.76 (d, 1H, *J* = 8.2 Hz, CH=), 5.45 (s, 2H, NCH_2_OBn), 4.66 (s, 2H, CH_2_Ph), 4.25 (d, 1H, *J* = 6.7 Hz, H-4), 4.15 (dd, 1 H, *J* = 2.8, 4.8 Hz, H-2′), 4.10–4.06 (m, 2H, H-3′, H-4′), 3.89–3.80 (m, 3H, H-7, H-5′, CH_2_OC-5′), 3.72–3.62 (m, 3H, H-3, H-5′, CH_2_OC-5′), 3.64 (dd, 1H, *J* = 5.9, 11.1 Hz, H-8), 3.54 (d, 1H, *J* = 6.9 Hz, H-2), 3.51 (dd, 1H, *J* = 6.2, 11.1 Hz, H-8), 3.27–3.12 (m, 2H, NHCH_2_), 2.05–1.96 (m, 2H, CH_2_), 1. 69 (dd, 1H, *J* = 4.0, 9.0 Hz, H-1), 0.93 (dd, 1H, *J* = 5.8, 9.0, Hz, H-6), 0.79 (dd, 1H, *J* = 4.0, 5.8 Hz, H-6); ^13^C-NMR (125 MHz, CD_3_OD, δ_C_) 164.8 (C=O), 152.4 (C=O), 141.6 (CH=), 139.4 (Ar), 129.4 (2C, Ar × 2), 128.8 (Ar), 128.7 (2C, Ar × 2), 102.1 (CH=), 93.0 (C-1′), 84.0 (C-4′), 79.6 (C-4), 76.2 (C-3), 75.3 (C-3’), 73.2 (CH_2_Ph), 71.9 (C-7), 71.6 (NCH_2_OBn), 71.6 (C-5′ or CH_2_OCH_2_), 71.1 (CH), 70.4 (C-5’ or CH_2_OCH_2_), 65.8 (C-8), 61.0 (C-2), 45.8 (NHCH_2_), 35.9 (C-5), 27.3 (CH_2_), 19.8 (C-1), 9.0 (C-6). HRMS (ESI) *m/z* Calcd for (M − CF_3_COO^−^) C_28_H_40_N_3_O_11_: 594.2657. Found: 594.2651.

*2′,3′-O-Isopropylidene-5′-O-(4-hydroxylbutyl)-3-(benzyloxymethyl)uridine* (**24**). To a solution of **20** (0.89 g, 1.6 mmol) and 1,4-butanediol (**22**) (1.62 g, 19.4 mmol) in DMF (4 mL) at 0 °C was added NaH (128 mg, 3.2 mmol, 60% in mineral oil). The reaction was stirred at rt for 20 h and then H_2_O (10 mL) was added. The mixture was extracted with Et_2_O and the organic layer was washed with brine, dried (MgSO_4_) and concentrated. The resulting residue was purified by chromatography (EtOAc–Hexane 2:1) to give **24** (0.40 g, 52%) as a colorless oil. *R_f_* 0.16 (EtOAc–Hexane 2.5:1); $[\alpha {]}_{D}^{25}$ −3.7 (*c* 2.43, CH_2_Cl_2_); ^1^H-NMR (500 MHz, CDCl_3_, δ_H_) 7.53 (d, 1H, *J* = 8.1 Hz, CH=), 7.36–7.23 (m, 5H, Ar), 5.83 (s, 1H, H-1), 5.71 (d, 1H, *J* = 8.1 Hz, CH=), 5.59 (d, 1H, *J* = 9.8 Hz, NCH_2_OBn), 5.45 (d, 1H, *J* = 9.8 Hz, NCH_2_OBn), 4.78–4.74 (m, 2H, H-2, H-3), 4.70 (s, 2H, CH_2_Ph), 4.38 (ddd, 1H, *J* = 2.6, 2.6, 3.9 Hz, H-4), 3.69 (dd, 1H, *J* = 2.6, 10.7 Hz, H-5), 3.61–3.57 (m, 3H, H-5, CH_2_OH), 3.59–3.46 (m, 2H, CH_2_), 1.64–1.55 (m, 4H, CH_2_CH_2_), 1.57 (s, 3H, CH_3_), 1.36 (s, 3H, CH_3_); ^13^C-NMR (125 MHz, CDCl_3_, δ_C_) 162.7 (C=O), 150.9 (C=O), 139.7 (CH=), 137.9 (Ar), 128.3 (2C, Ar × 2), 127.6 (3C, Ar × 3), 114.0 (C(CH_3_)_2_), 101.4 (CH=), 94.0 (C-1), 85.9 (C-4), 85.4 (C-2), 80.9 (C-3), 72.3 (CH_2_Ph), 71.6 (CH_2_OCH_2_), 70.7 (C-5), 70.3 (NCH_2_OBn), 62.4 (CH_2_OH), 29.5 (CH_2_), 27.2 (CH_3_), 26.2 (CH_2_), 25.4 (CH_3_). HRMS (ESI) *m/z* Calcd for (M + Na^+^) C_24_H_32_N_2_O_8_: 499.2051. Found: 499.2046.

*2′,3′-O-Isopropylidene-5′-O-(4-oxobutyl)-3-(benzyloxymethyl)uridine* (**26**). A solution of **24** (73 mg, 0.15 mmol) in CH_2_Cl_2_ (4 mL) was added to a solution of Dess-Martin periodinane (78 mg, 0.18 mmol) in CH_2_Cl_2_ (8 mL) at 0 °C. The reaction was stirred for 3 h at 0 °C and poured into an ice cold saturated aqueous NaHCO_3_ solution. The organic layer was washed with H_2_O, brine, dried (MgSO_4_) and concentrated. The residue was purified by chromatography (EtOAc–Hexane 1:1) to give **26** (60 mg, 83%) as a colorless oil. *R_f_* 0.28 (EtOAc–Hexane 2:1); $[\alpha {]}_{D}^{25}$ +17.1 (*c* 0.70, CH_2_Cl_2_); ^1^H-NMR (400 MHz, CDCl_3_, δ_H_) 9.72 (dd, 1H, *J* = 1.5, 1.5 Hz, CHO), 7.44 (d, 1H, *J* = 8.2 Hz, CH=), 7.43–7.24 (m, 5H, Ar), 5.80 (d, 1H, *J* = 2.0 Hz, H-1), 5.73 (d, 1H, *J* = 8.2 Hz, CH=), 5.50 (d, 1 H, *J* = 9.7 Hz, NCH_2_OBn), 5.48 (d, 1 H, *J* = 9.7 Hz, NCH_2_OBn), 4.78–4.74 (m, 2H, H-3, H-2), 4.71 (s, 2H, CH_2_Ph), 4.35 (ddd, 1H, *J* = 3.0, 4.4, 4.4 Hz, H-4), 3.67 (dd, 1H, *J* = 4.4, 10.7 Hz, H-5), 3.59 (dd, 1H, *J* = 4.4, 10.7 Hz, H-5), 3.53–3.45 (m, 2H, CH_2_), 2.50–2.46 (m, 2H, CH_2_), 1.92–1.87 (m, 2H, CH_2_), 1.59 (s, 3H, CH_3_), 1.37 (s, 3H, CH_3_); ^13^C-NMR (125 MHz, CDCl_3_, δ_C_) 201.4 (CHO), 162.6 (C=O), 150.9 (C=O), 139.7 (CH=), 137.9 (Ar), 128.3 (2C, Ar × 2), 127.6 (3C, Ar × 3), 114.2 (C(CH_3_)_2_), 101.6 (CH=), 94.2 (C-1), 85.9 (C-4), 85.2 (C-2), 80.7 (C-3),72.3 (CH_2_Ph), 70.8 (CH_2_OBn), 70.6 (C-5), 70.3 (CH_2_OCH_2_), 40.6 (CH_2_), 27.2 (CH_3_), 25.3 (CH_3_), 22.2 (CH_2_). HRMS (ESI) *m/z* Calcd for (M + Na^+^) C_24_H_30_N_2_O_8_: 497.1894. Found: 497.1894.

*(1R,2S,3S,4S,5S)-N-(4-(2’,3’-O-Isopropylidene-3-(benzyloxymethyl)uridin)-butyl)-5-(1,2-dihydroxyethyl)-3,4-dihydroxybicyclo[3.1.0]hexan-2-ammonium acetate* (**28**). To a mixture of **2** (7.2 mg, 0.038 mmol) and **26** (18 mg, 0.038 mmol) in freshly distilled CH_3_OH (2 mL) at 0 °C was added BH_3_·pyridine (20 mL, 0.16 mmol) and phosphate buffer (0.1 M, pH 6.8, 0.4 mL). The mixture was stirred overnight at rt and then acidified with HOAc to pH 5 before being concentrated. The residue was purified by chromatography on C_18_ silica gel (H_2_O–CH_3_OH 10:1→1:10) to give the product **28** (10 mg, 37%) as a white foam. *R_f_* 0.11 (Pure CH_3_OH; C_18_ silica gel TLC); $[\alpha {]}_{D}^{25}$ +10.9 (*c* 1.00, CH_3_OH); ^1^H-NMR (500 MHz, CD_3_OD, δ_H_) 7.74 (d, 1H, *J* = 8.1 Hz, CH=), 7.31–7.22 (m, 5H, Ar), 5.80 (d, 1H, *J* = 1.4 Hz, H-1’), 5.72 (d, 1H, *J* = 8.1 Hz, CH=), 5.46 (s, 2H, NCH_2_OBn), 4.81 (m, 2H, H-2′, H-3′), 4.66 (s, 2H, CH_2_Ph), 4.39–4.37 (m, 1H, H-4′), 4.17 (d, 1H, *J* = 6.8 Hz, H-4), 3.74 (dd, 1H, *J* = 6.3, 6.4 Hz, H-7), 3.69 (dd, 1H, *J* = 3.1, 10.7 Hz, 1 × CH_2_OCH_2_), 3.65 (dd, 1H, *J* = 6.4, 11.2 Hz, H-8), 3.61–3.57 (m, 2H, H-3, 1 × CH_2_OCH_2_), 3.50–3.47 (m, 3H, 2 × H-5’, 1 × H-8), 2.91 (d, 1H, *J* = 6.2 Hz, H-2), 2.73 (ddd, 1H, *J* = 6.2, 7.7, 11.6 Hz, NHCH_2_), 2.56 (ddd, 1H, *J* = 6.4, 7.6, 11.6 Hz, NHCH_2_), 1.61–1.49 (m, 5H, H-1, CH_2_CH_2_), 1.54 (s, 3H, CH_3_), 1.35 (s, 3H, CH_3_), 0.68–0.67 (m, 2H, 2 × H-6); ^13^C-NMR (125 MHz, CD_3_OD, δ_C_) 164.9 (C=O), 152.3 (C=O), 142.4 (CH=), 139.5 (Ar), 129.4 (2C, Ar × 2), 128.7 (Ar), 128.6 (2C, Ar × 2), 114.8 (C(CH_3_)_2_), 101.7 (CH=), 95.7 (C-1′), 87.6 (C-4′), 86.6 (C-2’), 82.7 (C-3′), 79.9 (C-4), 77.5 (C-3), 73.3 (CH_2_Ph), 73.2 (C-7), 72.2 (C-5’ or CH_2_OCH_2_), 71.9 (NCH_2_OBn), 71.6 (C-5’ or CH_2_OCH_2_), 66.0 (C-8), 60.6 (C-2), 48.7 (NHCH_2_), 34.7 (C-5), 28.3 (CH_2_), 27.5 (CH_3_), 26.9 (CH_2_), 25.5 (CH_3_), 23.5 (C-1), 8.9 (C-6). HRMS (ESI) *m/z* Calcd for (M − CH_3_COO^−^) C_32_H_46_N_3_O_11_: 648.3127. Found: 648.3135.

*(1R,2S,3S,4S,5S)-N-(4-(3-(Benzyloxymethyl)uridin)butyl)-5-(1,2-dihydroxyet-hyl)-3,4-dihydroxybicyclo[3.1.0]hexan-2-ammonium 2,2,2-trifluoroacetate* (**30**). Compound **28** (13 mg, 0.019 mmol) was dissolved in TFA (2 mL) at 0 °C, stirred at rt for 12 h and then concentrated. The resulting residue was purified by chromatography on Iatrobeads (CH_2_Cl_2_–CH_3_OH 10:1→1:3) to give **30** (12 mg, 88%) as a white foam. *R_f_* 0.19 (CH_3_OH–NH_4_OH 40:1); $[\alpha {]}_{D}^{25}$ +16.0 (*c* 0.15, CH_3_OH); ^1^H-NMR (400 MHz, CD_3_OD, δ_H_) 7.90 (d, 1H, *J* = 8.2 Hz, CH=), 7.34–7.22 (m, 5H, Ar), 5.83 (d, 1H, *J* = 2.9 Hz, H-1′), 5.74 (d, 1H, *J* = 8.2 Hz, CH=), 5.45 (s, 2H, NCH_2_OBn), 4.66 (s, 2H, CH_2_Ph), 4.27 (d, 1H, *J* = 6.9 Hz, H-4), 4.13–4.09 (m, 3H, H-2′, H-3′, H-4′), 3.90–3.80 (m, 3H, H-7, H-3, H-5′), 3.67–3.39 (m, 6H, H-5′, 2 × H-8, H-2, CH_2_OC-5′), 3.20–3.07 (m, 2H, NHCH_2_), 1.87–1.69 (m, 5H, CH_2_CH_2_, H-1), 0.94 (dd, 1H, *J* = 5.8, 8.7 Hz, H-6), 0.79 (dd, 1H, *J* = 4.0, 5.8 Hz, H-6); ^13^C-NMR (100 MHz, CD_3_OD, δ_C_) 164.9 (C=O), 152.5 (C=O), 141.3 (CH=), 139.4 (Ar), 129.4 (2C, Ar × 2), 128.8 (Ar), 128.7 (2C, Ar × 2), 101.9 (CH=), 92.3 (C-1′), 84.4 (C-4′), 79.6 (C-4), 76.3 (C-3), 75.8 (C-3’), 73.2 (CH_2_ Ph), 71.9 (C-7), 71.7 (CH_2_OC-5′), 71.6 (C-5’), 71.1 (C-2′), 70.9 (NCH_2_OBn), 65.8 (C-8), 60.8 (C-2), 47.0 (NHCH_2_), 35.9 (C-5), 27.8 (CH_2_), 24.3 (CH_2_), 19.9 (C-1), 9.1 (C-6). HRMS (ESI) *m/z* Calcd for (M–CF_3_COO^−^) C_29_H_42_N_3_O_11_: 608.2814. Found: 608.2809.

*(1R,2S,3S,4S,5S)-5-(1,2-Dihydroxyethyl)-N-((2S,3R)-2,3-O-isopropylidene-4-(2′,3′-O-iso-propylidene-5′-thiouridin)-2,3-dihydroxylbutyl)-3,4-dihydroxybicyclo[3.1.0]hexan-2-ammonium acetate* (**37**). A solution of **36** (30 mg, 0.067 mmol) in CH_2_Cl_2_ (4 mL) was added to a solution of Dess-Martin periodinane (29 mg, 0.067 mmol) in CH_2_Cl_2_ (8 mL) at 0 °C. The solution was stirred for 3 h and then poured into an ice cold saturated aqueous NaHCO_3_ solution. The organic layer was washed with H_2_O, brine, dried (MgSO_4_), and concentrated. The resulting aldehyde was dissolved in freshly distilled CH_3_OH (2 mL), and was added to **2** (12.7 mg, 0.067 mmol). The mixture was stirred for 1 h, cooled to 0 °C, and then BH_3_·pyridine (20 mL, 0.16 mmol) and phosphate buffer (0.1 M, pH 6.8, 0.4 mL) were added. After stirring overnight at rt, the solution was acidified with HOAc to pH 5 and then concentrated. The resulting residue was purified by chromatography on Iatrobeads (CH_2_Cl_2_–CH_3_OH 10:1→1:10) to give **37** (10 mg, 34%) as a white foam. *R_f_* 0.25 (Pure CH_3_OH); $[\alpha {]}_{D}^{25}$ +13.9 (*c* 1.28, CH_3_OH); ^1^H-NMR (400 MHz, CD_3_OD, δ_H_) 7.65 (d, 1H, *J* = 8.1 Hz, CH=), 5.74 (d, 1H, *J* = 2.3 Hz, H-1′), 5.69 (d, 1H, *J* = 8.1 Hz, CH=), 5.05 (dd, 1H, *J* = 2.3, 6.6 Hz, H-2′), 4.79 (dd, 1H, *J* = 4.1, 6.6 Hz, H-3′), 4.25–4.21 (m, 2H, H-4′, H-4), 4.05–3.97 (m, 1H, OCH), 3.96–3.91 (m, 1H, OCH), 3.82 (dd, 1H, *J* = 6.1, 6.3 Hz, H-7), 3.70 (dd, 1H, *J* = 6.4, 6.7 Hz, H-3), 3.66 (dd, 1H, *J* = 6.1, 11.1 Hz, H-8), 3.50 (dd, 1H, *J* = 6.3, 11.1 Hz, H-8), 3.19–3.15 (m, 2H, H-2, 1 × NHCH_2_), 2.97–2.95 (m, 2H, 2 × H-5′), 2.88–2.80 (m, 3H, 1 × NHCH_2,_ CH_2_S), 1.59 (dd, 1H, *J* = 4.3, 8.6 Hz, H-1), 1.53 (s, 3H, CH_3_), 1.40 (s, 3H, CH_3_), 1.39 (s, 3H, CH_3_), 1.34 (s, 3H, CH_3_), 0.77 (dd, 1H, *J* = 5.8, 8.6 Hz, H-6), 0.72 (dd, 1H, *J* = 4.3, 5.8 Hz, H-6); ^13^C-NMR (125 MHz, CD_3_OD, δ_C_) 166.1 (C=O), 151.9 (C=O), 144.8 (CH=), 115.5 (C(CH_3_)_2_), 110.9 (C(CH_3_)_2_), 103.0 (CH=), 95.3 (C-1′), 88.5 (C-4′), 85.5 (C-2′), 84.7 (C-3′), 80.3 (C-4), 80.2 (OCH), 80.0 (OCH), 77.4 (C-3), 72.9 (C-7), 66.1 (C-8), 60.9 (C-2), 51.4 (NHCH_2_), 36.1 (SCH_2_), 35.7 (SCH_2_), 34.9 (C-5), 27.6 (CH_3_), 27.5 (2C, 2 × CH_3_), 25.5 (CH_3_), 23.0 (C-1), 8.9 (C-6). HRMS (ESI) *m/z* Calcd for (M − CH_3_COO^−^) C_27_H_42_N_3_O_11_S: 616.2534. Found: 616.2525.

*(1R,2S,3S,4S,5S)-5-(1,2-Dihydroxyethyl)-N-((2S,3R)-4-(2’,3’-O-isopropylidene-5’-thiouridin)-2,3-dihydroxybutyl)-3,4-dihydroxybicyclo[3.1.0]hexan-2-ammonium 2,2,2-trifluoroacetate* (**31**). Compound **37** (10 mg, 0.015 mmol) was dissolved in TFA (2 mL) at 0 °C and the solution was then stirred at rt for 12 h and concentrated. The residue was purified by chromatography on Iatrobeads (CH_2_Cl_2_–CH_3_OH 10:1→1:3) to give the **31** (3 mg, 33%) as a white foam. *R_f_* 0.3 (CH_3_OH–NH_4_OH 20:1); $[\alpha {]}_{D}^{25}$ +23.2 (*c* 0.34, CH_3_OH); ^1^H-NMR (400 MHz, D_2_O, δ_H_) 7.73 (d, 1H, *J* = 8.1 Hz, CH=), 5.90 (d, 1H, *J* = 8.1 Hz, CH=), 5.86 (d, 1H, *J* = 4.2 Hz, H-1′), 4.40 (dd, 1H, *J* = 4.2, 4.6 Hz, H-2′), 4.34 (d, 1H, *J* = 7.2 Hz, H-4), 4.20–4.18 (m, 2H, H-3′, H-4′), 4.03 (ddd, 1H, *J* = 3.1, 3.1, 9.0 Hz, H-c), 3.94–3.91 (m, 2H, H-3, H-7), 3.81–3.75 (m, 2H, H-b, H-8), 3.57 (d, 1H, *J* = 6.4 Hz, H-2), 3.50 (dd, 1H, *J* = 7.7, 11.6 Hz, H-8), 3.26 (dd, 1H, *J* = 3.1, 12.7 Hz, H-d), 3.13–3.02 (m, 2H, H-d, H-5′), 2.95 (dd, 1H, *J* = 6.7, 14.2 Hz, H-5’), 2.86 (dd, 1H, *J* = 5.0, 13.8 Hz, H-a), 2.78 (dd, 1H, *J* = 8.2, 13.8 Hz, H-a), 1.71 (dd, 1H, *J* = 4.1, 8.9 Hz, H-5), 0.92 (dd, 1H, *J* = 5.6, 8.9 Hz, H-6), 0.84 (dd, 1H, *J* = 4.1, 5.6 Hz, H-6); ^13^C-NMR (100 MHz, D_2_O, δ_C_) 166.6 (C=O), 151.9 (C=O), 142.4 (CH=), 102.7 (CH=), 90.5 (C-1′),83.1(C-4′), 77.9 (C-2′), 75.1 (C-3′), 73.3, 72.2, 71.7, 71.3, 63.9, 59.5 (C-2), 49.4 (C-d), 35.3 (C-5), 34.0 (SCH_2_), 33.1 (SCH_2_), 23.5 (C-1), 8.5 (C-6); HRMS (ESI) *m/z* Calcd for (M − CF_3_COO^−^) C_21_H_34_N_3_O_11_S: 536.1909. Found: 536.1906.

### 3.2. General Methods for GlfT2 Inhibition Assays

Solutions of 2 M KCl, 1 M MgCl_2_, and 1 M 3-(*N*-Morpholino)propanesulfonic acid (MOPS) (pH 7.6) were prepared in deionized distilled (MilliQ, MQ) water, filtered, and stored at 4 °C. Recombinant GlfT2, prepared and stored as previously reported were used in the assay [29]. On the day of experiment, donor analogues were reconstituted in filtered MQ water to give a 32 mM stock. Solutions of 15 mM β-nicotinamide adenine dinucleotide (NADH), 5 U/mg Pyruvate Kinase (PK), 16.8 U/mg lactate dehydrogenase (LDH), and 40 mM UDP-Gal*f* were prepared in 50 mM MOPS (pH 7.6); 100 mM phosphoenolpyruvic acid monocyclohexylammonium salt (PEP) was prepared in 250 mM MOPS (pH 7.6); 40 mM trisaccharide (β-d-Gal*f*-(1→5)-β-d-Gal*f*-(1→6)-β-d-Gal*f*-Octyl) was prepared in filtered MQ water. All solutions were stored on ice during use.

### 3.3. Evaluation for Ability of Compounds to Serve as Inhibitors of GlfT2

Reactions to screen the ability of analogues to inhibit GlfT2 were initiated with the addition of GlfT2 (0.5 μg) to assays to give a final volume of 40 μL containing 50 mM MOPS (pH 7.6), 50 mM KCl, 20 mM MgCl_2_, 1.1 mM NADH, 3.5 mM PEP, 7.5 U PK, 16.8 U LDH, 2 mM trisaccharide, 4 mM analogues, and 0.75 mM donor UDP-Gal*f*. Reactions were incubated at 37 °C and monitored at 340 nm at 10–15 s intervals for 20 min using a Spectra Max 340 PC microplate reader. The inhibition screening assays were repeated at two-times linking enzyme levels (15 U PK and 33.6 U LDH), to rule out inhibition of the linking enzymes by the analogues.

## 4. Conclusions

In this paper we have described the preparation of a small library of UDP-Gal*f* mimetics based upon the bicyclo[3.1.0]hexane derivative 2. The key step in generating the products was the reductive amination between the amino group in 2 and aldehydes. In some cases, protecting group issues led to some of the desired targets not being accessible. Evaluation of these compounds analogues against the mycobacterial galactosyltransferase GlfT2 revealed that most of the compounds were poor inhibitors of the enzyme and, moreover, inclusion of the uridine moiety did not enhance inhibitory binding.
